# Dedicated Followers of Fashion? Bioarchaeological Perspectives on Socio‐Economic Status, Inequality, and Health in Urban Children from the Industrial Revolution (18th–19th C), England

**DOI:** 10.1002/oa.2531

**Published:** 2016-05-31

**Authors:** S. L. Newman, R. L. Gowland

**Affiliations:** ^1^ Department of ArchaeologyDurham UniversityDurhamUK

**Keywords:** post‐medieval, growth, palaeopathology, vitamin D deficiency, weaning, London

## Abstract

The 18th and 19th centuries in England were characterised by a period of increasing industrialisation of its urban centres. It was also one of widening social and health inequalities between the rich and the poor. Childhood is well‐documented as being a stage in the life course during which the body is particularly sensitive to adverse socio‐economic environments. This study therefore aims to examine the relationship between health and wealth through a comprehensive skeletal analysis of a sample of 403 children (0–17 years), of varying socio‐economic status, from four cemetery sites in London (c.1712–1854).

Measurements of long bone diaphyseal length, cortical thickness, vertebral neural canal size, and the prevalence of a range of pathological indicators of health stress were recorded from the Chelsea Old Church (high status), St Benet Sherehog (middle status), Bow Baptist (middle status), and Cross Bones (low status) skeletal collections.

Children from the low status Cross Bones site demonstrated deficient growth values, as expected. However, those from the high status site of Chelsea Old Church also demonstrated poor growth values during infancy. Fashionable child‐care practices (e.g. the use of artificial infant feeds and keeping children indoors) may have contributed to poor infant health amongst high status groups. However, differing health risks in the lower status group revealed the existence of substantial health inequality in London at this time. © 2016 The Authors International Journal of Osteoarchaeology Published by John Wiley & Sons Ltd.

## Introduction

The industrial cities of the 18th and 19th centuries in England were notorious for their unhealthy living conditions. Urban districts increasingly experienced severe overcrowding, poor sanitation and ventilation, and high levels of air pollution (Kay, 1832; Engels, 1950; Hudson, 1992). During this period, environmental conditions in London were notoriously poor: the capital was an ‘engine of growth’ of the industrial revolution and epitomised the ‘evils of the urban environment’ (O'Brien & Quinault, 1993: 236).

The expanding cities posed significant health risks, particularly for the youngest members of society. Children are vulnerable to adverse environments, because of their under‐developed immune system and the physiological demands of growth (Lewis, 2007; Cameron & Bogin, 2012). Under conditions of poor nutrition or infection, resources are diverted away from growth towards those functions necessary for immediate survival, resulting in growth deficits (Bogin, 1999; Sapolsky, 2004). Historical records from the 18th and 19th centuries attest to the common‐place stunting of growth, with children of the poorer classes most affected (Floud *et al.,* 1990; Sharpe, 2012).

This study assesses growth and skeletal indicators of stress in the skeletal remains of non‐adults (0–17 years) excavated from cemeteries known to comprise different social status groups from London (c.1712–1856). The aim is to discern whether health stress was experienced in children throughout all levels of the social strata, or whether children of middle to high status groups were buffered through social privilege from the detrimental effects of urban life.

## Health and the social gradient in the 18th/19th centuries

London was a highly stratified society during the 18th and 19th centuries: the rich and the poor lived in relatively close proximity (Booth, 1891), but would have experienced markedly different lives. At the upper echelons were the ‘gentry’ and the aristocracy, next came the ‘middling sort’, which included merchants, manufacturers, and skilled craftsmen who could afford a ‘comfortable’ living for their families (Earle, 1989; Crawford, 2010). At the lowest levels of the social ladder were the labouring families and the truly destitute, who were often forced to seek poor relief in the workhouses (Hudson, 1992; Crawford, 2010). Opportunities for social mobility were limited and children of the poor were likely to remain in poverty throughout their lives (Crawford, 2010: 5).

Social inequality is known to have a profound effect on the overall well‐being of a population, producing a social gradient in health that deteriorates as income lessens (Marmot & Wilkinson, 2006; Wilkinson & Pickett, 2010; Pickett & Wilkinson, 2015). Social inequalities are associated with low birth‐weight, higher rates of infant mortality, shorter height, poor health, and lower life expectancies (Marmot & Wilkinson, 2006; Wilkinson & Pickett, 2010; Elgar *et al.,* 2015). Cultural practices such as child‐care strategies also have implications for child health, and these are known to be influenced by social status today (Oakley *et al.,* 2014), and class differentiated child‐care practices were also present during the period in question. It has been suggested that social gradients in health may not have existed prior to the late‐19th to early‐20th century (Razzell & Spence, 2006). Therefore, it is of great interest to determine whether the bioarchaeological analysis of skeletal growth and pathology reveals evidence of social inequalities in non‐adult health within this study.

## Materials and methods

The four skeletal collections analysed in this study are summarised in Table 1. Data for the sexes were pooled, as there is currently no reliable method of determining sex prior to skeletal maturity (Saunders, 2008). Overall, 403 non‐adult skeletons (0–17 years of age) were assessed for evidence of pathology, and measurements of growth were taken where preservation allowed.

**Table 1:** Summary of sites located in London

Site	Date	Status	No. individuals	No. non‐adults	% non‐adults within sample
Chelsea Old Church	1712–1842	High	198	33	16.7
St Benet Sherehog	<1853	Middle	230	64	27.8
Bow Baptist	1816–1856	Middle	416	202	48.6
Cross Bones	1800–1853	Low	148	104	70.3

**Figure 1:**
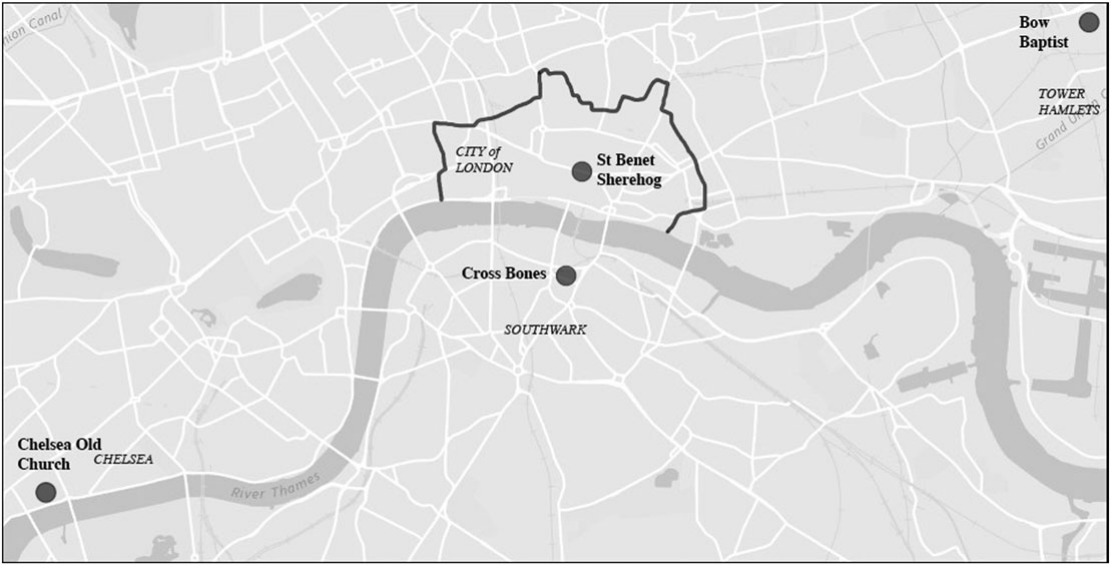
Locations of cemeteries within London. Grey line delimits old city boundaries established during Roman occupation, which continued to represent the centre of London throughout the expansion of the city during the 18th/19th centuries.

The sample of Chelsea Old Church represents individuals of a higher status who resided in the suburbs of the city (see Figure 1). By the mid‐18th century Chelsea still retained an almost rural status, and has been described as ‘a relatively healthy and prosperous place’ and ‘a fashionable resort for Londoners’ (Cowie *et al.,* 2008: 13). However, as London continued to grow, by the mid‐19th century it had become engulfed by the urban sprawl (Cowie *et al.,* 2008).

The St Benet Sherehog and Bow Baptist skeletal samples represent individuals of the ‘middling’ classes. St Benet Sherehog church was located within the inner city of London (see Figure 1), and this parish has been described as affluent and small (Miles *et al.,* 2008a). This site was in use prior to the Great Fire in 1666, and continued to be used as a burial ground up until its closure in the mid‐19th century (Miles *et al.,* 2008a). Therefore, while the post‐medieval sample from the St Benet Sherehog collection is representative of individuals interred in the post‐Fire burial ground, it is possible that some individuals within this collection may date to an earlier period. The Bow Baptist group resided within the small village of Bow on the outskirts of the city at the beginning of 1816 (Henderson *et al.,* 2013). However, the rapid industrialisation of London in the second half of the 19th century quickly subsumed this community into the expanding metropolis (Henderson *et al.,* 2013).

Last, the Cross Bones cemetery was an unconsecrated burial ground located in the parish of St Saviour's, Southwark (Figure 1), and was reserved for the very poorest of society (Brickley & Miles, 1999). This skeletal collection represents those who would have experienced the most squalid levels of sanitation, with areas of the parish being described as ‘a ruinous and filthy slum’ (Brickley & Miles, 1999: 20).

Dental age, appositional growth, and vertebral growth data were collected from each sample by the lead author. Detailed pathological descriptions from the Wellcome Osteological Research Database (WORD) were also reassessed in line with recent publications (see below) to construct crude prevalence rates of pathology.

### Age assessment

Dental age was assessed using standards for dental calcification (Smith, 1991) of both the deciduous and permanent dentition, for comparability with previous growth studies (Pinhasi *et al.,* 2006). Calcification stages were determined by examination of the dentition radiographically, or macroscopically when loose teeth were present.

### Longitudinal growth

Tibial lengths for individuals within each sample were plotted against dental age to assess longitudinal growth (a proxy for growth in height in non‐adults). The tibia was selected because it is considered to be more sensitive than the other long bones to growth disruption (Bogin *et al.,* 2002; Pomeroy *et al.,* 2012). The maximum diaphyseal length of the left tibia (substituted with the right side when necessary) was measured using a standard osteometric board, according to the standards of Buikstra & Ubelaker (1994). Tibial length had previously been recorded for the four collections according to the above standards by the Museum of London (MoL), and was provided by the WORD database (WORD database, 2012a, 2012b, 2012c), and the Museum of London Archaeology (MoLA) for the Bow Baptist collection. Tibial lengths of ‘healthy’ children (from Denver, Colorado, US) aged 0–18 years of age were taken from the study by Maresh (1955), to provide a modern comparative data‐set.

### Appositional growth

Appositional growth (growth in the width of long bones) was assessed through measurement of cortical thickness. Cortical thickness (CT) is considered to be a more sensitive indicator of stress than longitudinal growth (Mays *et al.,* 2009). The left femur was selected for CT measurement (substituted with the right side when necessary), to ensure comparability with the previous study by Mays *et al.* (2009). Radiographs were taken using a Kubtec Xtend 100HF x‐ray source and Kubtec 3600 CR reader at 54 kVp and 5 mAs, with a 120‐cm source‐image receptor‐distance (SID) (Gerald Conlogue, personal communication 20th April 2015). Measurements of the total bone width (T) and the medullary width (M) were taken from the mid‐shaft of the femur (Mays *et al.,* 2009). CT was determined as T‐M, and plotted against the dental age. Modern comparative data for femoral CT was provided by the study by Virtama & Helelä (1969) of ‘healthy’ Finnish children (0–18 years of age).

### Vertebral growth

Vertebral neural canal (VNC) size is an effective indicator of stress in early life (Watts, 2013a, 2013b). This skeletal feature completes the majority of growth during the first 2 years of age; therefore any disruption to growth occurring prior to this becomes ‘locked‐in’ (Scheuer & Black, 2000; Watts, 2013b). Measurements of transverse diameter in children can be used to form vertebral growth profiles, with the mid‐thoracic region of the vertebral column demonstrating the least inherent variation (see Newman & Gowland, 2015 for methodology). Measurements of transverse diameter of the neural canal of vertebrae T6–8 were taken using sliding calipers (to the nearest 0.01 mm). Averages of the vertebral measurements from T6 to 8 were calculated for each individual and then plotted against dental age to form growth profiles. The study by Hinck *et al.* (1966) provided modern comparative data (from Oregon, US) for transverse diameter for both children (0–18 years) and adults (18+ years).

### Statistical analysis

Scatterplots were constructed for tibial length, femoral CT, and TR diameter to determine the homogeneity of the regression slopes (Field, 2013). Once it had been confirmed that all assumptions had been met, the data were statistically assessed via analysis of covariance (ANCOVA). This statistical test allows for the comparison of the regression slopes of two datasets, while acknowledging the influence of dental age as a covariate (Pinhasi *et al.,* 2006; Field, 2013). This method was only applied to individuals between 0 and 12 years of age, because of the influence of the sex‐differentiated pubertal growth spurt in adolescence (Lewis *et al.,* 2015).

### Pathology

The presence or absence of four pathological categories was determined using data collected from the WORD database (WORD database, 2012a; WORD database, 2012b; WORD database, 2012c; data for the Bow Baptist collection provided by MoLA). The crude prevalence rate (CPR) for rickets, scurvy, periosteal new bone formation, and dental enamel hypoplasia within each site was calculated as a percentage of individuals demonstrating signs of each pathology within the sample.

As the diagnostic criteria for rickets and scurvy have developed over recent years, all individuals were re‐categorised according to recent publications (Brickley & Ives, 2008; Armelagos *et al.,* 2014; Klaus, 2014a; Stark, 2014) into the groups ‘scurvy’, ‘possible scurvy’, ‘rickets’, ‘possible rickets’, and ‘metabolic disease’ based on detailed descriptions from the WORD database. The ‘metabolic disease’ category includes all individuals demonstrating skeletal changes indicative of ‘rickets’, ‘possible rickets’, ‘scurvy’, ‘possible scurvy’, and those that could not be reliably placed into either of the aforementioned categories. This ensured that prevalence of metabolic disease could be assessed with due consideration to the issues associated with misdiagnosis of rickets and scurvy, and also their frequent co‐morbidity (Stark, 2014).

Classic indicators of rickets in the skeleton include bowing of the long bones, flaring of the metaphyses, and thickening of the diaphyses (Mays *et al.,* 2006; Pinhasi *et al.,* 2006). Other indicators include porosity of the growth plate, new bone formation, flaring of the rib ends, and porosity of the cranial bones (Ortner & Mays, 1998; Brickley & Ives, 2008). The category ‘rickets’ includes all cases indicative of rickets, and possible rickets (i.e. when more subtle manifestations of some of the above characteristics were observed).

Scurvy, resulting from vitamin C deficiency, can be detected skeletally through the presence of new bone formation and porosity resulting from haemorrhaging of the blood vessels in areas where movement frequently occurs; for example, in the orbits, on long bones (in association with the joints), and on the mandible and maxillae (Brickley & Ives, 2008; Armelagos *et al.,* 2014; Klaus, 2014a). Other skeletal indicators include new bone formation and porosity on the cranial bones and scapulae (Brickley & Ives, 2006; Armelagos *et al.,* 2014; Klaus, 2014a). The category ‘scurvy’ includes all cases indicative of scurvy, and possible scurvy.

Periosteal new bone formation refers to areas of bone formation, or porosity on the cranial bones and long bones that cannot be attributed to a specific cause (Weston, 2008; Klaus, 2014b). This indicator was recorded as present when evidence of new bone formation (woven and/or lamellar) was identified on the long bones or ectocranial surface of the cranial bones. No attempt was made to assess coverage or severity, and no differentiation between healed/unhealed lesions was made.

Dental enamel hypoplasia (DEH), arising because of the disturbance of enamel formation in the developing teeth (Hillson, 2008), was recorded as present when one or more teeth demonstrated evidence of linear or pitted defects. Malnutrition and episodes of disease have been identified as major influences in the occurrence of DEH (Ogden *et al.,* 2007; Hillson, 2008).

The overall sample sizes for pathological analysis, longitudinal growth, appositional growth, and vertebral growth are provided in Table 2.

**Table 2:** Sample sizes for pathological and metric analysis (adult sample size)

Site	Pathology	Tibial length	Femoral CT	Vertebral TR (T6–8)
Chelsea Old Church	33	10	10	10
St Benet Sherehog	64	9	13	8
Bow Baptist	202	70	48	46
Cross Bones	104	36	37	22

## Results

### Age‐at‐death

**Figure 2:**
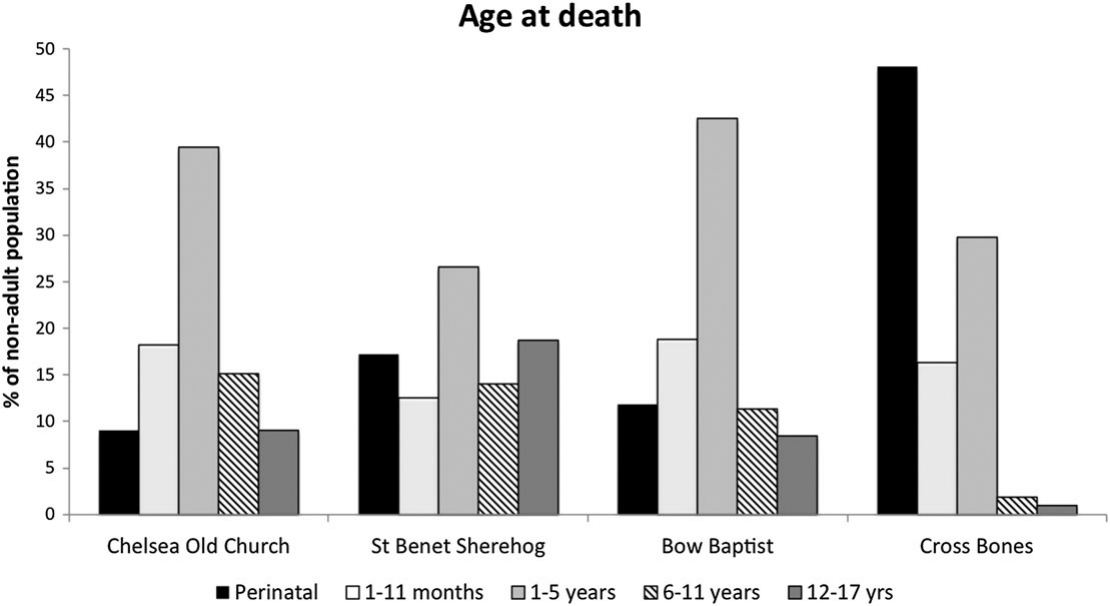
Proportion of non‐adults within each group falling into the age categories perinatal (approximately 36 weeks in utero to 4 weeks post‐partum), 1–11 months, 1–5 years, 6–11 years, and 12–17 years. Percentages for each sample based on the total number of non‐adults for each site.

Figure 2 shows the age‐at‐death distribution of the sample. The peak age‐at‐death for the Chelsea Old Church, St Benet Sherehog, and Bow Baptist samples is 1–5 years of age (at 39%, 27%, and 43%, respectively). However, for Cross Bones the perinatal age group represents nearly half of the non‐adult sample (at 48%).

### Longitudinal growth

**Figure 3:**
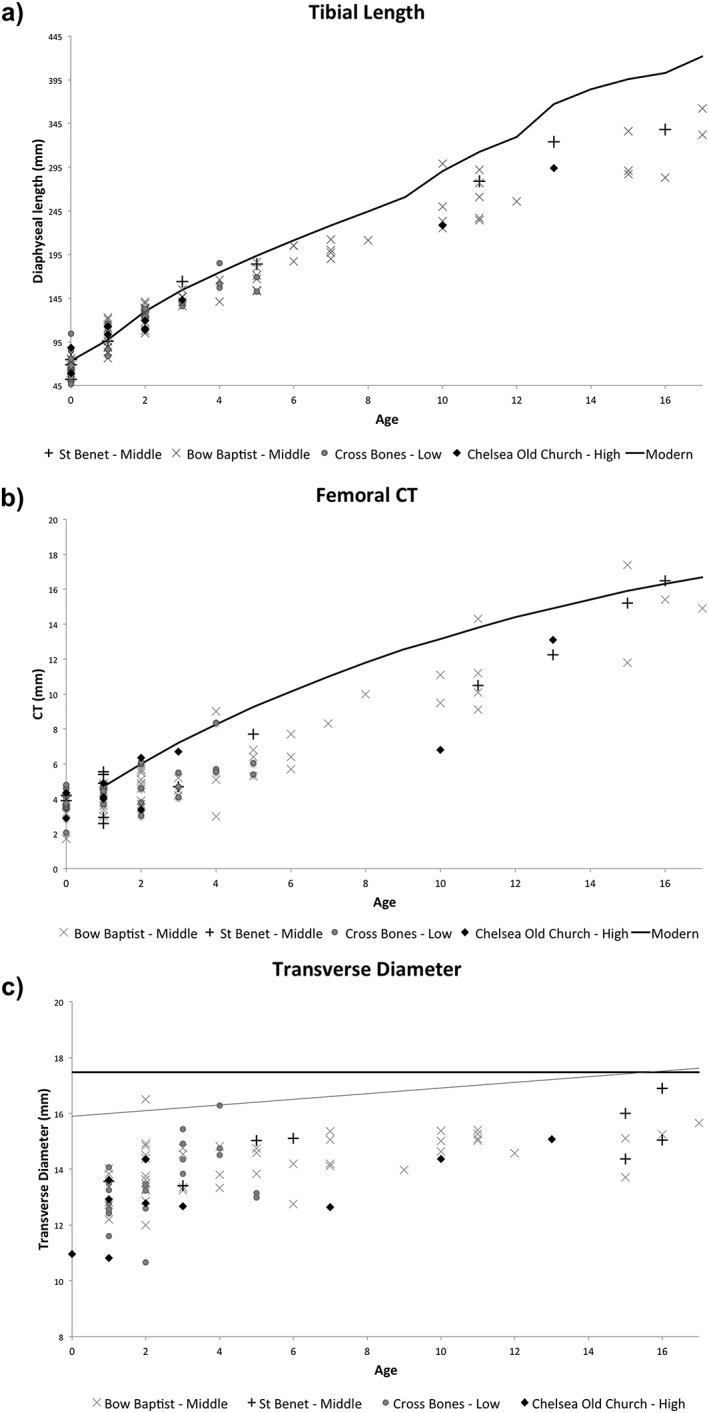
Growth profiles for skeletal parameters plotted against dental age: a) tibial length—comparative modern data represented by solid black line, taken from Maresh (1955); b) femoral CT—comparative modern data represented by solid black line, taken from Virtama & Helelä (1969); c) transverse diameter for T6–8 comparative modern adult data (solid black line) and modern non‐adult data (solid grey line) taken from Hinck *et al.* (1966).

Tibial lengths were plotted against dental age, with each data point representing one individual (Figure3a). All sites show growth comparable to the modern data for the first 2 years of life. However, from approximately 2–5 years of age all of the archaeological groups fall below the modern values. While St Benet Sherehog displays some of the highest values throughout the remainder of the growth period, Chelsea Old Church exhibits some of the lowest up until 10 years of age. Beyond 10 years this sample then appears to ‘catch‐up’; however, this may be an artefact of the small sample size of this site. It is of note that by 17 years of age, none of the Chelsea Old Church, St Benet Sherehog, and Bow Baptist samples have reached modern values for tibial length. At 16 years of age the St Benet Sherehog and Bow Baptist samples have only reached approximately 84% and 70% of the modern values respectively. Unfortunately, the Cross Bones data‐set for tibial length does not extend beyond 5 years of age because of preservation. There were no statistically significant differences between the tibial lengths of the four archaeological sites (Table 3). However all archaeological samples were significantly lower than the modern data‐set (Table 3).

**Table 3:** ANCOVA results for measurements of tibial length, femoral CT, and transverse diameter (TR) from the four archaeological samples, and compared to modern data. *P* ≤ 0.05, significant values in bold. Modern data taken from Maresh (1955), Virtama & Helelä (1969), and Hinck *et al.* (1966)

		Tibial length		Femoral CT		TR	
		F	*p*	F	*p*	F	*p*
Chelsea Old Church vs	St Benet Sherehog	0.106	0.750	0.334	0.571	4.444	0.061
	Bow Baptist	0.415	0.522	0.007	0.934	10.420	**0.002**
	Cross Bones	0.111	0.741	0.021	0.885	3.236	0.083
St Benet Sherehog vs	Chelsea Old Church	0.106	0.750	0.334	0.571	4.444	0.061
	Bow Baptist	0.000	0.999	0.527	0.471	0.455	0.504
	Cross Bones	0.010	0.919	0.470	0.497	0.132	0.720
Bow Baptist vs	Chelsea Old Church	0.415	0.522	0.007	0.934	10.420	**0.002**
	St Benet Sherehog	0.000	0.999	0.527	0.471	0.455	0.504
	Cross Bones	3.234	0.075	0.338	0.563	1.045	0.311
Cross Bones vs	Chelsea Old Church	0.111	0.741	0.021	0.885	3.236	0.083
	St Benet Sherehog	0.010	0.919	0.470	0.497	0.132	0.720
	Bow Baptist	3.234	0.075	0.338	0.563	1.045	0.311
Modern vs	Archaeological	5.313	**0.001**	11.957	**0.000**	6.453	**0.000**

### Appositional growth

Figure 3b shows the femoral measurements for CT plotted against dental age. The majority of individuals from all of the archaeological sites fall below the modern comparative data‐set, revealing statistically significant deficiencies (Table 3). This deviation away from the modern data occurs from approximately 1–3 years onwards. Individuals from Chelsea Old Church demonstrate some notable deficiencies, particularly in the first 2 years of life, and at 10 years of age, before attaining higher values once again by 13 years. St Benet Sherehog and the Bow Baptists attain similar values to that seen in the modern population by 15 years of age, reaching approximately 100% and 94% of the modern data values. Individuals from Cross Bones show a mix of higher and lower values for CT at birth and in infancy; however, between 2 and 5 years of age these remain particularly low. Again no data for CT exists beyond 5 years of age for Cross Bones. There are no statistically significant differences between the four archaeological groups (Table 3).

### Transverse diameter

Measurements of transverse diameter for each individual were plotted against dental age (Figure 3d). All of the archaeological data sets are severely deficient in comparison to the modern data, and this is statistically significant (Table 3). St Benet Sherehog is the closest to reaching modern values. Chelsea Old Church and Cross Bones have the lowest values for transverse diameter in the first 2 years, averaging approximately 72% and 73% of the modern adult values respectively. Chelsea Old Church continues to demonstrate deficient values for the remainder of the growth period, only achieving approximately 86% of the modern adult values by 13 years of age, and demonstrates significantly lower values than the Bow Baptists (Table 3).

### Prevalence of pathology

**Figure 4:**
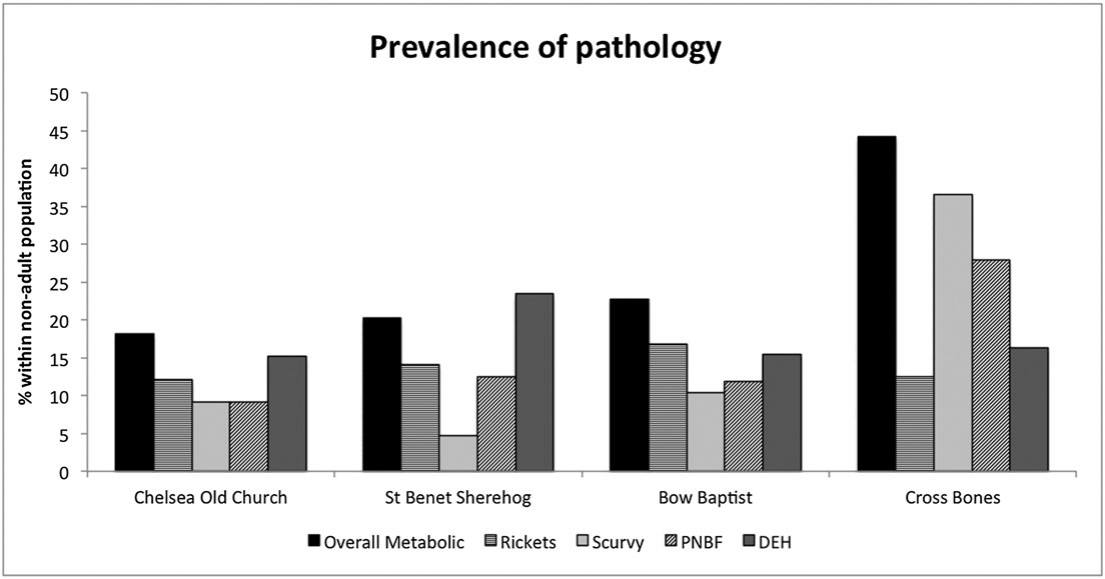
Crude prevalence rate (CPR) of pathology seen in each skeletal sample. PNBF = periosteal new bone formation; DEH = dental enamel hypoplasia.

All four groups have a high rate of rickets, between approximately 12% and 17% (Figure 4). The overall rate of metabolic disease is highest in Cross Bones, at approximately 44% compared to 18–23% at the other sites. This is because of the extremely high rate of scurvy in this group, affecting 37% of the non‐adult sample. The rate of scurvy is much lower in the other sites, particularly St Benet Sherehog (at 5%). The prevalence rate of periosteal new bone formation is also much higher within Cross Bones (28%), compared to only 9%, 13%, and 12% of non‐adult individuals in the Chelsea Old Church, St Benet Sherehog, and Bow Baptist samples respectively. The prevalence of DEH is similar between the Chelsea Old Church, Bow Baptist, and Cross Bones groups (15%, 13%, and 16%). However it is most prevalent in the St Benet Sherehog sample (23%). Overall, while prevalence of pathology is high in all of the archaeological groups, Cross Bones shows the highest rate of skeletal pathologies.

## Discussion

### Evidence for infant care

Kay stated in 1832 that ‘…more than one‐half of the offspring of the poor…die before they have completed their fifth year’ (p.42–43), and around 42% of total deaths in London between 1837 and 1838 were in those aged 5 years and under (Registrar‐General, 1839). All four sites also demonstrated a preponderance of young children aged between 1 and 5 years. High rates of DEH were observed across each site, in addition to statistically significant deficiencies in growth when compared to modern data. This deviation from the modern data occurred in general between 1 and 5 years of age for tibial length and femoral CT (Figures 4a and 4b). This is corroborated by the particularly deficient values for the transverse diameter (which completes the majority of its growth by 2 years of age) in all of the skeletal samples (Jinkins, 2000; Scheuer & Black, 2000). From these results, it can be inferred that individuals from each of the archaeological samples suffered insults to health in infancy, regardless of social class.

Overall, the non‐adults of St Benet Sherehog showed the highest growth values for tibial length, femoral CT, and transverse diameter. However, this could reflect the more favourable environmental conditions prior to the 18th century, as some individuals from this site may date to the 17th century. Cross Bones and Chelsea Old Church demonstrate some of the lowest values for all growth parameters, indicating particularly poor health in infancy. While these results were expected for the low status site of Cross Bones, the results for the wealthier population of Chelsea Old Church were surprising.

High rates of metabolic disease were evident in all of the archaeological samples, with all four groups demonstrating similar rates of rickets. The occurrence of metabolic disease peaks between 1 and 5 years for Chelsea Old Church and St Benet Sherehog, and between 1 and 11 months and 1–5 years of age for the Bow Baptist sample (Table 4). Rickets and scurvy during this period were particularly associated with ‘weanlings’ (those aged 6 months–2 years) (Crawford, 2010). Therefore, while metabolic disease occurring in perinates at the Cross Bones sample is likely to be related to maternal health, the later peaks in prevalence within the other sites may relate to an early onset of weaning and/or a deficient weaning diet.

**Table 4:** Breakdown of metabolic disease by age category. Metabolic disease referring number of individuals within the skeletal sample demonstrating any sign of rickets, possible rickets, possible scurvy, and scurvy. Percentage in brackets

Site	No. non‐adults	Perinatal	1–11 months	1–5 years	6–11 years	12–17 years	Unknown	Overall
Chelsea Old Church	33	0 (0)	0 (0)	3 (9.1)	1 (3)	2 (6.1)	0 (0)	6 (18.2)
St Benet Sherehog	64	2 (3.1)	1 (1.6)	6 (9.4)	1 (1.6)	0 (0)	3 (4.7)	13 (20.3)
Bow Baptist	202	4 (2)	18 (8.9)	21 (10.4)	2 (1)	1 (0.05)	0 (0)	46 (22.8)
Cross Bones	104	24 (23.1)	11 (10.6)	11 (10.6)	0 (0)	0 (0)	0 (0)	46 (44.2)

During the 18th and 19th centuries breastfeeding fluctuated in popularity, and upper class parents tended not to take a ‘hands‐on’ role in the rearing of children, who were instead left in the care of nursemaids (Burnett, 1984; Perkin, 1993). Amongst the higher classes, breastfeeding was viewed as unfashionable or inconvenient (Perkin, 1993; Stevens *et al.,* 2009). Consequently, artificial feeding or wet nurses were popular alternatives (Wickes, 1953; Fildes, 1995; Nitsch *et al.,* 2011). By contrast, in lower class families resources were often stretched because of inconsistent employment, low wages, and increasing family size (Burnett, 1984). Working‐class mothers often prioritised allocation of food resources within the family to the ‘bread‐winners’, frequently leading to malnourishment in mothers and younger children (Humphries, 2010; Horrell & Oxley, 2012). Expectant mothers in employment would continue to work up to 18 h a day until birth, returning soon afterwards (as little as three days) to ensure continued employment (Engels, 1950; Perkin, 1993). This often meant accelerating the process of weaning, and complete cessation of breastfeeding from an early age (Wickes, 1953; Fildes, 1995; Nitsch *et al.,* 2011). The high levels of infection and malnutrition amongst the lower classes also meant that many mothers would have been unable to produce nutritionally adequate breast milk (Cheadle, 1889; Fildes, 1995). Conversely, poorer families in which the mother did not work may have chosen to prolong the breastfeeding period, as it would have been a cheaper alternative to providing food to an additional member of the family (Crawford, 2010; Nitsch *et al.,* 2011). However, beyond six months of age the nutritional constituents of breast milk alone are not sufficient to support the energetic needs of the growing infant (Haggerty & Rutstein, 1999).

The immature immune system of the newborn infant relies on the transmission of maternal antibodies via breast milk for protection from environmental pathogens, and development of their own immunocompetance (Cunningham, 1995; Katzenberg et al., 1996). Clinical studies have observed that infants fed food other than breastmilk suffer from more frequent bouts of acute illnesses (Stuart‐Macadam & Dettwyler, 1995; Haggerty & Rutstein, 1999; Ip *et al.,* 2007; Horta & Victoria, 2013). Therefore, it is likely that the status‐driven breastfeeding strategies practiced in the 18th and 19th centuries had a significant impact on infant health.

An isotopic study by Nitsch *et al.* (2011) revealed a variety of feeding practices from the middle class skeletal sample from Christ Church, Spitalfields, London (which was contemporaneous with the sites here), ranging from infants receiving little to no breastmilk, to those breastfed until around 1.5 years of age. Popular artificial infant feeds at this time included ‘paps’ and ‘panadas’ prepared from flour or bread mixed with water, or cow's milk (Wickes, 1953; Rendall, 1990; Drummond & Wilbraham, 1994). Infants can only absorb around 10% of the iron available in cow's milk and this, alongside the starchy food, could have caused irritation of the immature digestive tract and resulted in iron‐deficiency anaemia (Stuart‐Macadam & Dettwyler, 1995; Lewis, 2002). Cow's milk was also a source of bacterial diseases such as scarlet fever and tuberculosis (Rendall, 1990; Atkins, 1992; Drummond & Wilbraham, 1994).

Additionally, higher status infants were frequently swaddled: wrapped in strips of material to protect them from cold, and to help their limbs grow straight (Cadogan, 1748; Buchan, 1778; Rousseau, 1889). Children of the middling and upper classes were also often kept indoors to protect them from ‘moral or physical contamination’ (Burnett, 1984: 48), and direct sunlight was believed to be bad for babies' eyesight (Drummond & Wilbraham, 1994). These child‐care practices and infant feeding strategies meant that higher‐class children would have been susceptible to developing deficiencies in vitamin D, especially in infancy. Increased rates of rickets within high‐status families have been noted in previous studies (Miles *et al.,* 2008b; Giuffra *et al.,* 2013). Given the importance of vitamin D for immune response (Holick, 2003), the high rates of rickets amongst the wealthier children of London, alongside diminished immunocompetance from the use of breastmilk substitutes, would have left these infants highly susceptible to the polluted urban environment.

### Evidence for social inequalities in health

The low status group of Cross Bones was dominated by perinates. The skeletal sample represents only a small proportion of the entire cemetery, which was not completely excavated, and so the predominance may represent sample bias (Brickley & Miles, 1999). However, burial registers for the parish of St Saviour's (where the cemetery was located) reveal that of the 270 burials in 1845 approximately 45% were those aged 16 years and below, with the majority of deaths occurring between 0 and 1 years of age (Brickley & Miles, 1999). This high rate of perinatal death likely reflects the harsher environmental conditions, heightened exposure to early life stressors, and poor maternal health associated with being born into poverty. This corroborates the results of a study by DeWitte *et al.* (2015), which also identified heightened mortality risks for children of lower status from 18th/19th century London‐based skeletal collections.

Overall, Cross Bones demonstrates the highest prevalence of pathology. Peaks in metabolic disease occurred from the perinatal stage, to 1–5 years of age (Table 4). Particularly notable is the high rate of scurvy, occurring primarily in perinates (Table 4). Young infants should not develop scurvy, as vitamin C is provided via maternal diet while *in utero*, or breastmilk following birth (Cheadle, 1889; Brickley & Ives, 2008). In order for perinates to be affected, maternal health must have been severely compromised, demonstrating the inter‐generational consequences of poverty for health. This finding is confirmed by the high rates of DEH in the deciduous dentition of Cross Bones (at 14% of the non‐adult sample). The deciduous dentition commences development *in utero*; therefore, these defects again reflect maternal health stress. Cross Bones likewise demonstrated some of the lowest growth values for tibial length and femoral CT in infancy, and some of the greatest deficiencies in transverse diameter (reflecting disruption in the first 2 years of life). Poor maternal health may lead to intrauterine growth restriction, resulting in infants that are born small‐for‐gestational age (SGA) (Eveleth & Tanner, 1990; Hernandez‐Beltran *et al.,* 1996). These early growth deficits may lead to permanent stunting, and mothers who were born SGA themselves are much more likely to produce SGA infants (Haeffner *et al.,* 2002; Prentice *et al.,* 2006). Therefore, the effects of poor environment on birth size may span multiple generations (Hernandez‐Beltran *et al.,* 1996).

Studies by Barker (1992, 1994) and Barker & Osmond (1986) have revealed the significant impact that infection and nutrition during pregnancy and infancy can have on future adult health, termed the Developmental Origins of Health and Disease (DOHaD) hypothesis. Maternal malnutrition limits the nutrition available to the foetus, with longer‐term implications for the development of physiological and metabolic responses (Barker, 1992, 1994; Scott & Duncan, 2000). These changes in physiology increase chronic disease risk in later life, such as coronary heart disease, chronic bronchitis, stomach cancer, and diabetes (Barker & Osmond, 1986; Barker, 1992, 1994; Wadsworth & Butterworth, 2006). Hughes‐Morey (2015) found that, when compared to high status females from Chelsea Old Church, low status females from St Bride's Lower, London, demonstrated a lower mean age‐at‐death, and significantly shorter femora (Hughes‐Morey, 2015). Watts (2015) also identified an association between deficiencies in adult TR diameter and a reduction in longevity within the Chelsea Old Church, St Benet Sherehog, and Cross Bones collections. Paucities in health identified in the non‐adult samples in this study, combined with these previous studies, therefore demonstrate that early life stress in the 18th and 19th centuries had implications for future adult health.

Inequalities in health are thus often rooted in early life and can continue to impact on health throughout the life course (Elgar *et al.,* 2015). These initial health disadvantages can be exacerbated by postnatal care strategies because of the limited resources of families living in poverty, highlighting the importance of both prenatal (maternal) environment and postnatal exogenous influences (such as environmental conditions and social factors) on future health and well‐being (Aizer & Currie, 2014).

All of the samples in this study were affected by high infant mortality and morbidity, but the lower status Cross Bones sample was most severely affected. While it is true that Chelsea Old Church also demonstrated some of the lowest growth values alongside Cross Bones, it must be considered that these deficiencies developed for very different reasons. Children of wealthier families were more likely to succumb to poor health because of fashionable child care practices, whereas mothers born and raised in poverty could pass on their health deficits to their children. This, combined with the detrimental external environment and disadvantageous family economy, meant that life chances at birth for the children of the poor were limited to a high risk of premature death, or a lifetime of poor health and hard labour (Gowland, 2015; Gowland & Newman, in press).

While interesting patterns related to child health and social status in the 18th/19th centuries have been identified, it is important to consider the limitations that accompany studies such as these. The current issues surrounding the identification of sex in non‐adult individuals creates a limiting factor in the study of growth within adolescents, because of sex‐related differences in the pubertal growth spurt. Without knowing the proportion of males and females within an adolescent sample, it is not known whether the data is skewed towards higher or lower growth values for a particular age. Sample size is also an inherent issue in bioarchaeology. The requirement for well‐preserved skeletal elements for measurement often means that sample sizes are small. Additionally, differential preservation of skeletal elements between populations means that, as seen in this study, sample sizes of collections may be imbalanced. The use of modern data as a comparative sample to infer whether past skeletal populations were ‘unhealthy’ by present standards is also problematic, as this involves comparing those who did not survive childhood, and were thus inherently ‘weaker’, to healthy living children.

## Conclusion

Life in the city came with some significant health risks to all of its members. While undoubtedly the lower classes experienced a much higher risk of mortality and morbidity, this study revealed that some of these risks were also often felt by the upper classes, supporting recent research by DeWitte *et al.* (2015), Hughes‐Morey (2015), and Watts (2015). Poor infant health was aggravated by child‐care practices heavily influenced by a family's position within society, being dictated either by the desire to follow fashion amongst the upper echelons, or necessity amongst the poor. These promoted conditions such as rickets and scurvy, and led to high infant mortality that spanned the social strata. Amongst the poor, the inter‐generational consequences of poverty were apparent in the high rates of perinatal death, but also in the intrauterine onset of deficiency diseases.
